# Stellate ganglion block attenuates chronic stress induced depression in rats

**DOI:** 10.1371/journal.pone.0183995

**Published:** 2017-08-31

**Authors:** Weiwei Wang, Weidong Shi, Hua Qian, Xijin Deng, Tong Wang, Wenzhi Li

**Affiliations:** 1 Department of Anaesthesiology, Second Affiliated Hospital of Harbin Medical University, Harbin, China; 2 Center for Endemic Disease Control, Chinese Center for Disease Control and Prevention, Harbin Medical University, Harbin, China; Technion Israel Institute of Technology, ISRAEL

## Abstract

**Background:**

Stress is a significant factor in the etiology of depression. Stellate ganglion block (SGB) has been shown to maintain the stability of the autonomic system and to affect the neuroendocrine system, including the hypothalamic–pituitary–adrenal (HPA) axis. The objective of this study was to determine the antidepressant-like effects of SGB on the autonomic system and the HPA axis, apoptosis-related proteins, related spatial learning and memory impairment, and sensorimotor dysfunction.

**Methods:**

Forty-eight Sprague Dawley rats were assigned to four experimental groups: control + saline (sham group), control + SGB (SGB group), unpredictable chronic mild stress (UCMS) + saline (UCMS group), and UCMS + SGB (UCSG group). Stress-induced effects and the function of SGB were assessed using measures of body weight, coat state, sucrose consumption, and behavior in open-field and Y-maze tests. Neuronal damage was assessed histologically using the hematoxylin-eosin (HE) staining method, while western blotting was used to investigate changes in the expression of apoptosis-related proteins. Plasma corticotropin-releasing factor (CRF), adrenocorticotropic hormone (ACTH), corticosterone (CORT), noradrenaline and adrenaline were measured to evaluate changes in the autonomic system and HPA axis.

**Results:**

SGB treatment significantly improved sensorimotor dysfunction and spatial learning and memory impairment following UCMS. Moreover, UCMS significantly decreased body weight, sucrose preference and anti-apoptotic protein Bcl-2, and increased scores on measures of coat state, adrenal gland weight, levels of CORT, CRF, ACTH, noradrenaline and adrenaline, as well as increased neuronal loss, cell shrinkage, nuclear condensation, and the pro-apoptotic protein Bax. These symptoms were attenuated by treatment with SGB.

**Conclusions:**

These findings suggest that SGB can attenuate depression-like behaviors induced by chronic stress. These protective effects appear to be due to an anti-apoptotic mechanism of two stress pathways–the autonomic system and the HPA axis.

## Introduction

Depression, a series of disorders affecting many aspects of human physiology, is a public health problem with high morbidity and mortality rates [1]. It is also one of the costliest diseases in the European Union, where costs of affective disorders exceeded 113 billion euro in 2010 [2]. In the etiology of depression, stress is believed to be the most significant factor [3]. The stress response is an adaptive response by all living organisms to stressful events and is essential for survival. Changes in physiology induced by the stress response include the activation of the autonomic nervous system (overactivity of the sympathetic-adrenomedullary system) and the activation of multiple neuroendocrine axes [4, 5]. The hypothalamic-pituitary-adrenal (HPA) axis is one of the most important neuroendocrine axes. Hyperactivity of the HPA axis is considered to be a typical neurobiological alteration in depression [6,7]. These facts suggest that attenuating the overactivity of the HPA axis or hyperactivity of the sympathetic nervous system may contribute to the treatment of depression. Moreover, studies have demonstrated that apoptosis-related proteins, such as Bcl-2 and Bax, play an important role in neuronal death following chronic mild stress (CMS) [8, 9], which is also correlated with depression-like behavioral changes in rodents [10].

Currently, therapy for depression often involves different types of antidepressant drugs including tricyclic antidepressants, selective serotonin reuptake inhibitors, and monoamine oxidase inhibitors. However, the onset of appreciable clinical effects of antidepressant drugs is at least 3–4 weeks, also they can exert multiple adverse side effects and often result in unsatisfactory efficacy [11]. Taken together, the ongoing efforts to look for new antidepressant therapies remains an area of considerable interest.

Stellate ganglion block (SGB) is defined as the blockade of the sympathetic chain in the cervical and lower cervical and upper thoracic region. This is a commonly used technique for a variety of diagnostic, therapeutic and prognostic purposes [12]. It is well known that SGB is an effective therapy for patients with pain disorders. In addition, SGB has also been used, albeit somewhat controversially, in the management of various diseases without the feature of pain [13]. SGB has been shown to maintain the stability of the autonomic system through reversing the autonomic imbalance induced by increased sympathetic tone [14,15]. Moreover, SGB could also affect the neuroendocrine system (such as the HPA axis) by regulating the levels of several hormones and neuropeptides [16].

These findings prompted us to explore whether SGB treatment could attenuate changes to the autonomic system and the HPA axis, and prevent injury to neurons induced by CMS by altering Bcl-2 and Bax expression. The objective of this study was to evaluate the antidepressant-like effects of SGB on the autonomic system, HPA axis, apoptosis-related proteins, as well as spatial learning and memory impairment, and sensorimotor dysfunction in a chronic stress rat model.

## Materials and methods

### Experimental animals

Adult male Sprague-Dawley rats, weighing 180–240g, were obtained from the Animal Experiment Center of Harbin Medical University (Harbin, China). All experiments were approved by the Animal Ethics Committee of Harbin Medical University.

### Experimental protocol

**Fig 1:**
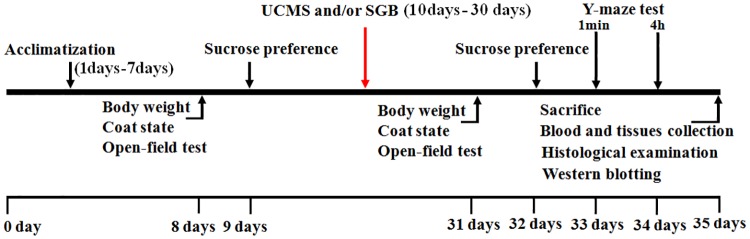
Experimental timeline for all procedures.

Rats were kept in cages (one per cage, with the exception of non-stressed animals) under controlled conditions (22 ± 2°C, 12 h light ⁄12 h dark cycle, except the reversed light-dark cycle periods in the unpredictable chronic mild stress [UCMS] procedure) and had free access to food and water at all times throughout the study. All rats were allowed to acclimatize in communal plastic cages for 7 days before treatment. After an acclimatization period of 1 week, the baseline determination of body weight, coat state, open-field test, and sucrose preference were performed over the subsequent two days. On day 10, following initial measurements, animals with similar baseline characteristics were randomly divided into four groups (n = 12): control + saline group (Sham group), control + SGB group (SGB group), UCMS + saline group (UCMS group), and UCMS + SGB group (UCSG group). Rats of the UCMS and UCSG groups, stress-treated animals, were exposed to the UCMS procedure for a period of 3 consecutive weeks. Animals from the other two non-stressed groups were housed under normal conditions in a separate room and had no contact with the stressed rats. Following the UCMS period, the stress-induced effects were assessed again by measuring body weight, coat state, sucrose consumption, and behavior in the open-field and Y-maze tests during the subsequent 4 days. On day 35, the rats were sacrificed under intraperitoneal injection of pentobarbital anesthesia. Blood and tissues of the brain and adrenal glands were quickly harvested and histological examination and western blot analysis were performed (see Fig 1 for experimental protocol). In addition, extraneous stressors, including interference from noise and people without fixed duties, were minimized and rat cages were cleaned regularly (except for rats exposed to UCMS) to remove suffering.

### Open-field test

The open-field test was performed as described by Blokland et al. [17], with modifications, in order to assess general locomotor activity. The testing apparatus consisted of a large black arena (100 cm × 100 cm × 30 cm) divided by strips into 25 equal-sized squares illuminated by white light. Each rat was placed individually in the center square of the apparatus. The time spent in the central zones, the number of squares traversed (where all four paws entering a square was regarded as an event), rearing (the frequency of standing on hind limbs) and grooming activity were recorded during a 5-min observation period.

### Stellate ganglion block (SGB)

In the present study, right SGB was used as a preventive therapeutic method to treat stress-induced effects, as described by Abdi et al. [18] with minor modifications. Rats received right SGB daily 1 h before being exposed to different stressors throughout the UCMS procedure. The animals were lightly anesthetized with isofluorane. The cartilaginous process of the C7 spinous process was palpated and a needle attached to a 1 ml syringe was inserted along the right side of the C7 vertebral body. When the tip of the needle lost contact with the vertebral body, it was withdrawn ~ 0.5 mm and the local anesthetic (0.2 ml of 0.25% bupivacaine hydrochloride injection, Shanghai He Feng Pharmaceutical Co. Ltd., Shanghai, China) was injected. Rats that were not treated with SGB received the same procedures as described previously, except that they were administered 0.2 ml of 0.9% physiological saline. All animals recovered from the general anesthesia within a few minutes. Right ptosis was recorded as an index of being blocked by SGB successfully.

### Unpredictable chronic mild stress (UCMS) procedure

The UCMS procedure used in our laboratory consisted of various social and environmental stressors, as previously described [19, 20], with slight modifications. The stress regime composed of the following elements: soiled cage (200 ml water in sawdust bedding) for 23 h, 45° cage tilt for 23 h, confinement to a small cage (8.7 cm in diameter) for 6 h, low intensity stroboscopic illumination (150 flashes/min) for 12 h, reversed light/dark cycle for 23 h, swimming in 4°C water for 5 min, 45°C heat stimulation for 5 min, paired housing for 2 h, and social stress (rat placed in another cage for 2 h and then returned to its own cage to find that it had been occupied by another rat). During the entire experiment, each stressor was repeated at least two times and was randomly administered to avoid adaptation by the rats.

### Evaluation of coat condition

Consistent with a previous study [21], the condition of the coat on different parts of the body, including the head, neck, ventral area, dorsal area, tail, front and hind paws, and genital area, was examined by an evaluator who was blind to group assignment. If the coat was in good condition or very poor condition (such as piloerection, disordered), the scores of 0 and 1 were applied, respectively, for each area. The total score was summed from the different areas on each rat, and a high score suggested that the coat was in poor condition.

### Sucrose preference test

At the end of the UCMS procedure, a sucrose preference test was employed to measure anhedonia, as previously described [20]. Animals were food and water deprived 24 h before the test, and then provided with the 1% sucrose solution and water for 24 h. Sucrose and water consumption were recorded by weighing the bottle containing the solution before and after the test.

### Y-maze test

The second day after having finished the sucrose preference test, the Y-maze 1 min test and Y-maze 4 h test were performed separately on the following 2 days to assess learning and spatial memory according to the method used by Conrad [22]. The apparatus consisted of three equilateral arms (58 cm × 19 cm × 38 cm) including one start arm and two alternate arms. Animals were first placed in the start arm for 15 min with the other two arms blocked. Rats were then removed from the maze, and then replaced into the Y-maze to freely explore all three arms for 5 min following either a 1 min or 4 h delay period. The number of entries into the three arms was recorded to assess short-term and long-term learning and spatial memory following UCMS and/or SGB treatments.

### Histological examination

The rats were decapitated, and the brains were removed, postfixed in 4% paraformaldehyde for 24 h, paraffin-embedded, sectioned at 3 μm, deparaffinized, hydrated sequentially, and processed for hematoxylin-eosin (HE) staining. Histological changes in the hippocampal CA3 area were observed using light microscopy (100 or 400 × magnification) by an evaluator who was blind to group assignment.

### Western blot analysis

Apoptosis-related proteins, Bcl-2 and Bax, were measured by western blot analysis (n = 5), as previously described [23]. Briefly, whole-cell lysates were obtained by homogenizing the tissue samples in a lysis buffer (5 mM EDTA, 50 mM Tris-HCl, 10 mM NaF, 150 mM NaCl, 0.5% sodium deoxycholate, 1 mM sodium orthovanadate, 1% Triton X-100, 1 mM phenylmethylsulfonyl fluoride, proteinase inhibitor cocktail tablets, and pH 7.5). The solubilized tissues were then centrifuged at 14,000 *g* at 4°C for 5 min and supernatants containing protein were collected for immunoblot analysis. The BCA Protein Assay Kit was used to determine the protein concentration. Equivalent amounts of protein (20–30 μg) were separated by sodium dodecyl sulfate-polyacrylamide gel electrophoresis on 10% polyacrylamide gels and then transferred to a nitrocellulose membrane. The membrane was blocked with 5% nonfat dry milk in a buffer of Tris-buffered saline with Tween (TBST) and incubated with respective primary antibodies for Bcl-2, Bax, and GAPDH (control) overnight at 4°C (Bcl-2 and Bax: 1:1000, Cell Signaling Technology Inc., Danvers, MA, USA; GAPDH: 1:1000, Santa Cruz Biotechnology, Heidelberg, Germany). After extensive rinsing in TBST buffer, the membranes were incubated with appropriate secondary antibodies (1:5000, Rockland Inc., Gilbertsville, PA, USA) for 1 h at room temperature, and developed with an enhanced chemiluminescence system (ECL Plus, Amersham Bioscience, Piscataway, NJ, USA). Percentage of sham (100%) was used to quantify the expression of protein.

### Determination of CRF, ACTH, corticosterone, noradrenaline, and adrenaline levels

Plasma corticotropin-releasing factor (CRF), adrenocorticotropic hormone (ACTH), corticosterone (CORT), noradrenaline, adrenaline were determined using enzyme-linked immunosorbent assay kits (R&D Systems, Minneapolis, MN, USA), according to the manufacturer’s instructions, respectively.

### Statistical analysis

All statistical analyses in this study were performed with SPSS 13.0 (version 13.01S; Beijing Stats Data Mining Co. Ltd, Beijing, China). Data are presented as mean ± standard error of mean (SEM). Repeated measures analysis of variance (ANOVA) followed by a Tukey’s post-hoc test was used to compare the difference in levels of body weight, coat state, open-field test, and sucrose preference. For analysis of the Y-maze test and the levels of plasma CRF, ACTH, CORT, noradrenaline, and adrenaline, one-way ANOVA was performed, followed by a Tukey’s post-hoc test. Bivariate Correlations with Spearman was used to analyze the correlation between behavioral indexes and hormonal levels. All P-values are two-tailed, and a *P*-value of < 0.05 was considered significant for all statistical analyses.

## Results

### Body weight and coat state changes

**Fig 2:**
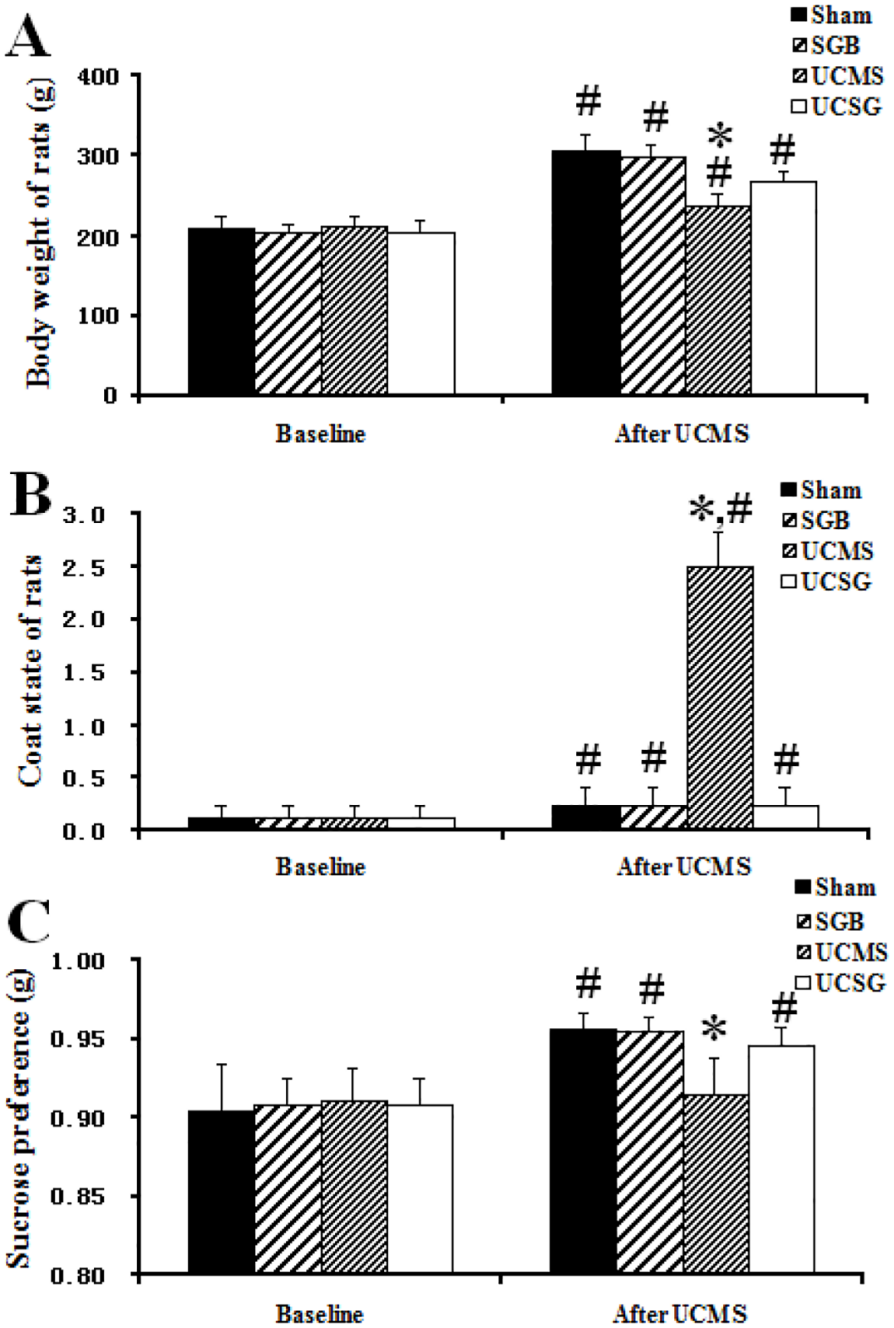
Behavioral phenotype and sucrose preference in rats. A, Overall coat state; B, Changes of body weight; C, Sucrose preference. *, *P* < 0.05 versus other groups after UCMS and/or SGB treatment; # *P* < 0.05 versus groups at baseline.

After 3-weeks of UCMS and/or SGB treatment(s), the average body weight of rats and coat state scores in all groups increased significantly compared with baseline, and there were interactions between the experimental time and UCM or SGB (before and after intervention in the Sham, SGB, UCMS and UCSG groups: body weight, 207.73 ±16.69 g vs. 305.73 ± 21.46 g, 202.82 ± 10.61 g vs. 298.53 ± 15.75 g, 213.35 g ± 10.40 g vs. 237.07 ± 14.88 g, and 204.25 g ± 14.27 vs. 268.87 ± 12.63 g; coat state, 0.12 ± 0.15 vs. 0.25 ± 0.16, 0.12 ± 0.15 vs. 0.25 ± 0.16, 0.12 ± 0.15 vs. 2.50 ± 0.33 and 0.12 ± 0.15 vs. 0.25 ± 0.16; *P* < 0.05). The average body weight of rats in the UCMS group was significantly lower and the coat state score was significantly higher than other groups (*P* < 0.05). SGB attenuated the decreased body weight and remarkably improved the score of coat state following chronic mild stress (Fig 2A and 2B).

### Sucrose preference test

The sucrose preference in Sham, SGB and UCSG groups increased significantly compared with baseline, and there was an interaction between the experimental time and UCMS (before and after intervention in Sham, SGB and UCSG groups: 0.91 ± 0.03 vs. 0.96 ± 0.01, 0.91 ± 0.02 vs. 0.96 ± 0.01, 0.91 ± 0.02 vs. 0.95 ± 0.01; *P* < 0.05). The CMS-exposed rats showed a decrease in sucrose preference in comparison with rats in the other groups (UCMS group vs. Sham, SGB and UCSG groups: 0.92 ± 0.02, vs. 0.96 ± 0.01, 0.96 ± 0.01, 0.95 ± 0.01, respectively, *P* < 0.05). This decrease was attenuated by treatment with SGB (Fig 2C).

### Open-field test analysis

**Fig 3:**
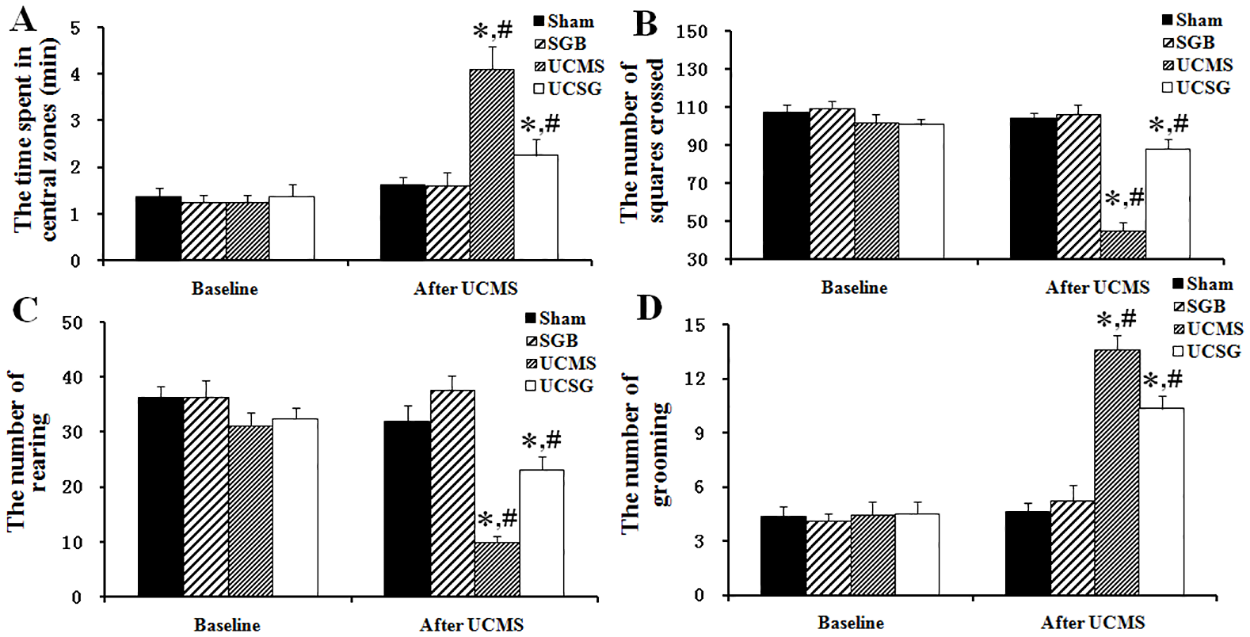
Open-field test scores in rats. A, Time in central zones; B, Squares crossed; C, Rearing frequency; D, Grooming frequency. *, *P* < 0.05 versus other groups after UCMS and/or SGB treatment; # *P* < 0.05 versus groups at baseline.

Fig 3 showed changes in locomotion and exploration in the open-field test. The time spent in the central zones and the grooming frequency increased significantly. Furthermore, rearing frequency and squares traversed decreased significantly in the UCMS and UCSG groups compared with the baseline. There were interactions between the experimental time and the UCMS or SGB treatments (before and after intervention in UCMS and UCSG groups: central zones, 1.25 ± 0.46 vs. 4.12 ± 0.98, 1.37 ± 0.54 vs. 2.25 ± 0.76; grooming, 4.50 ± 2.00 vs. 13.62 ± 2.82, 4.50 ± 1.92 vs. 10.37 ± 2.32; squares traversed, 102.25 ± 5.83 vs. 45.37 ± 4.09, 100.75 ± 4.61 vs. 88.00 ± 5.36; rearing, 29.37 ± 6.13 vs 10.00 ± 3.29, 32.37 ± 7.62 vs 23.12 ± 5.36, respectively, *P* < 0.05). After the 3-week CMS exposure, there was a significant increase in the time spent in the central zones and in grooming frequency, and a significant decrease in rearing frequency and squares traversed by rats in the UCMS group compared with rats in other groups (UCMS group vs. Sham, SGB and UCSG: central zones, 4.12 ± 0.98 vs. 1.62 ± 0.18, 1.62 ± 0.26, 2.25 ± 0.76; grooming, 13.62 ± 2.82 vs. 4.62 ± 3.36, 5.25 ± 5.46, 10.37 ± 2.32; squares traversed, 45.37 ± 4.09 vs. 103.87 ± 3.36, 106.12 ± 5.46, 88.00 ± 5.36; rearing, 10.00 ± 3.29 vs. 32 ± 3.36, 37.62 ± 5.46, 23.12 ± 5.36, respectively, *P* < 0.05). The increased time spent in the central zones and increased grooming frequency, decreased rearing frequency and decreased squares traversed induced by the CMS were all successfully attenuated by SGB treatment.

### Y-maze test analysis

**Fig 4:**
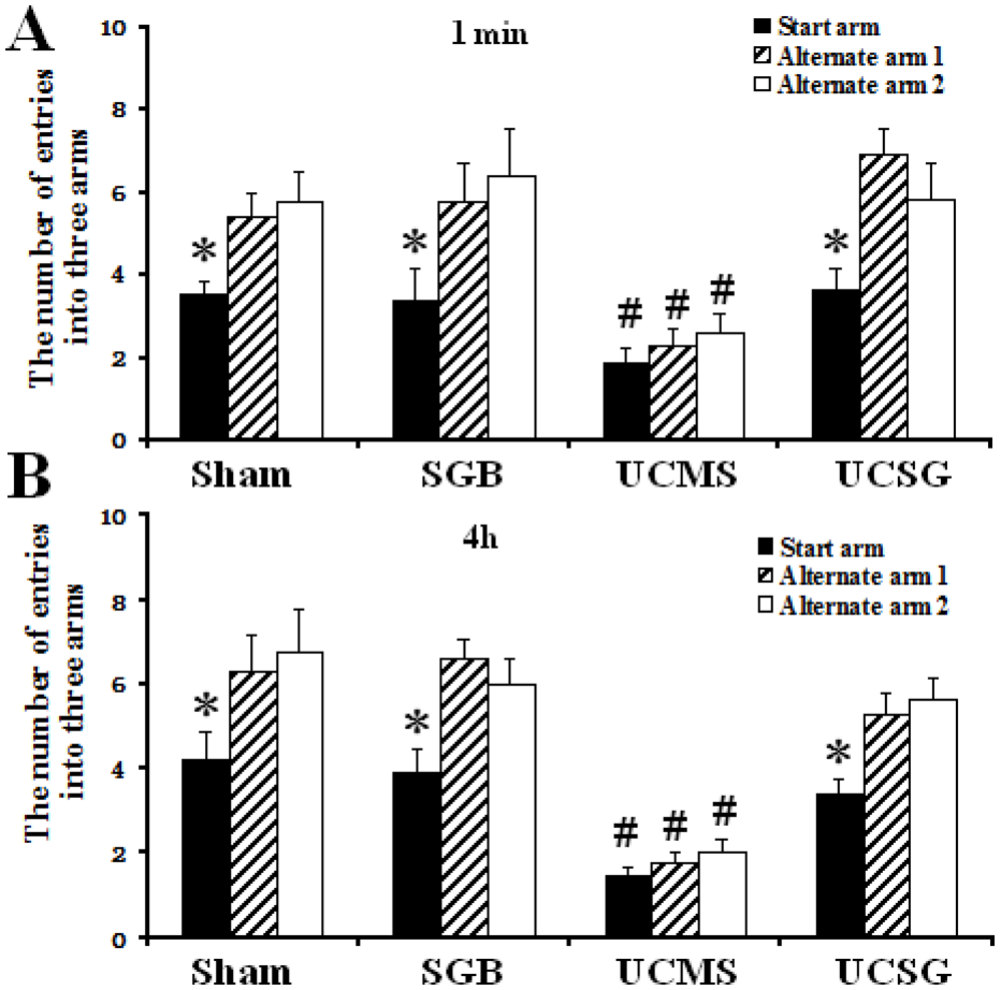
The number of entries into the three arms in the Y-maze test in rats. A and B, The number of entries into the three arms in 1-min and 4-h tests, respectively. *, In comparison with other arms, *P* < 0.05. # *P* < 0.05 versus other groups.

As shown in Fig 4, there was no significant difference in the number of entries into the three arms by rats in the UCSM group in either the 1 min or the 4 h Y-maze tests (for 1 min test, Start arm vs. Alternate arm 1 vs. Alternate arm 2: 1.88 ± 0.35, 2.25 ± 0.45, 2.63 ± 0.42, and for 4 h test, 1.50 ± 0.19, 1.75 ± 0.25, 2.00 ± 0.33). The rats in the other groups showed a higher number of entries into the alternative arms than the start arm (rats with normal brain function are willing to explore unfamiliar places). In the sham group, for 1 min test, Start arm vs. Alternate arm 1 and Alternate arm 2: 3.50 ± 0.33 vs. 4.625 ± 0.53 and 5.75 ± 0.75 and for 4 h test, 4.62 ± 0.63 vs. 5.62 ± 0.82 and 7.25 ± 1.01, *P* < 0.05; In SGB group, for 1 min test, Start arm vs. Alternate arm 1 and Alternate arm 2: 3.38 ± 0.80 vs. 4.87 ± 0.91 and 6.37 ± 1.15 and for 4 h test, 4.13 ± 0.55 vs. 4.87 ± 0.52 and 6.88 ± 0.64, *P* < 0.05; In UCSG group, for 1 min test, Start arm vs. Alternate arm 1 and Alternate arm 2: 3.62 ± 0.49 vs. 5.00 ± 0.65 and 7.25 ± 0.88 and for 4 h test, 3.38 ± 0.38 vs. 4.00 ± 0.46 and 5.63 ± 0.49, *P* < 0.05. The number of entries into the three arms by rats in the UCSM group was significantly lower than rats in the other groups in both the 1 min and the 4 h Y-maze tests. These results suggest that SGB treatment provides protective effects on short-term and long-term learning and spatial memory, and appears to attenuate the negative effects of UCMS.

### Adrenal gland weight change

The variations in adrenal gland weight are shown in Table 1. Following the 3-week CMS exposure, the adrenal gland weight in the UCMS group was significantly higher than that in other groups (*P* < 0.05). The results suggest that SGB treatment had beneficial effects on the CMS-exposed rats through attenuating the increase of adrenal gland weight.

**Table 1:** Differences of study variables among different groups.

	Sham	SGB	UCMS	UCSG
	(n = 12)	(n = 12)	(n = 12)	(n = 12)
**Left Adrenal weight (mg)**	16.34 ± 0.42	16.21 ± 0.46	23.73 ± 0.55 *	18.52 ± 0.41
**Plasma CRF (pmol/mL)**	43.68 ± 2.10	43.78 ± 2.49	67.18 ± 2.51 *	57.31 ± 3.72
**Plasma ACTH (pg/mL)**	48.60 ± 2.91	50.77 ± 3.38	70.61 ± 2.84 *	53.38 ± 3.16
**Plasma CORT (ng/mL)**	4.51 ± 0.21	4.89 ± 0.26	5.99 ± 0.14 *	4.99 ± 0.19
**Plasma noradrenaline (pg/mL)**	38.77 ± 2.34	40.52 ± 2.15	55.64 ± 3.83 *	44.75 ± 1.88
**Plasma adrenaline (ng/mL)**	15.26 ± 0.93	16.92 ± 0.88	21.46 ± 0.51 *	15.42 ± 0.62

Data are expressed as mean ± SEM; CRF, corticotropin releasing factor; ACTH, adrenocorticotropic hormone; CORT, corticosterone.

* *P* < 0.05 versus other groups.

### Changes in plasma CRF, ACTH, CORT, noradrenaline, and adrenaline following CMS exposure

Following the 3-week CMS exposure, the plasma levels of CORT, CRF, ACTH, noradrenaline, and adrenaline in the UCMS group were significantly higher than that of other groups (*P* < 0.05). The results suggest that CMS treatment increased the levels of CORT, CRF, ACTH, noradrenaline and adrenaline. SGB treatment ameliorated these increases (Table 1).

### Correlations between behavioral indexes and hormonal levels

There were significant positive correlations between hormone levels and coat state score, time spent in the central zones, and grooming frequency. Significant negative correlations were found between hormone levels and sucrose preference, squares traversed, and rearing frequency. Correlation analysis between the sucrose preference test and the open-field test analysis showed significant positive correlations between sucrose preference and squares traversed, and rearing frequency. Additionally, significant negative correlations were found between sucrose preference and time spent in the central zones, and grooming frequency. Among the behavior indexes, there were significant positive correlations among coat state scores, time spent in the central zone, and grooming frequency, and significant negative correlations between coat state scores, time spent in the central zone, grooming frequency with squares traversed, and rearing frequency (Table 2).

**Table 2:** Correlations analysis results.

	Correlation (*r*, *p*)									
	Sucrose preference	Time spent in the central zones	Squares traversed	Rearing frequency	Grooming frequency	CORT	CRF	ACTH	Noradrenaline	Adrenaline
**Coat state score**	-0.623, < 0.001	0.543, 0.001	-0.591, < 0.001	-0.618, < 0.001	0.465, 0.007	0.373, 0.035	0.642, < 0.001	0.437, 0.012	0.478, 0.006	0.332, 0.047
**Sucrose preference**		-0.546, 0.001	0.745, < 0.001	0.694, < 0.001	-0.700, < 0.001	-0.611, < 0.001	-0.755, < 0.001	-0.478, 0.006	-0.625, < 0.001	-0.419, 0.008
**Time spent in the central zones**			-0.490, 0.004	-0.591, < 0.001	0.514, 0.003	0.543, 0.001	0.691, < 0.001	0.53, 0.002	0.434, 0.013	0.350, 0.011
**Squares traversed**				0.745, < 0.001	-0.687, < 0.001	-0.575, 0.001	-0.611, < 0.001	-0.574, 0.001	-0.341, 0.056	-0.371, 0.026
**Rearing frequency**					-0.623, < 0.001	-0.445, 0.011	-0.619, < 0.001	-0.638, < 0.001	-0.474, 0.006	-0.398, 0.042
**Grooming frequency**						0.524, 0.002	0.627, < 0.001	0.475, 0.006	0.544, 0.001	0.396, 0.002
**CORT**							0.405, 0.021	0.385, 0.030	0.437, 0.012	0.337, 0.021
**CRF**								0.48, 0.005	0.683, < 0.001	0.426, 0.017
**ACTH**									0.329, 0.037	0.216, 0.029
**Noradrenaline**										0.331, 0.016

CRF, corticotropin releasing factor; ACTH, adrenocorticotropic hormone; CORT, corticosterone.

### Histological changes following UCMS exposure and SGB treatment

**Fig 5:**
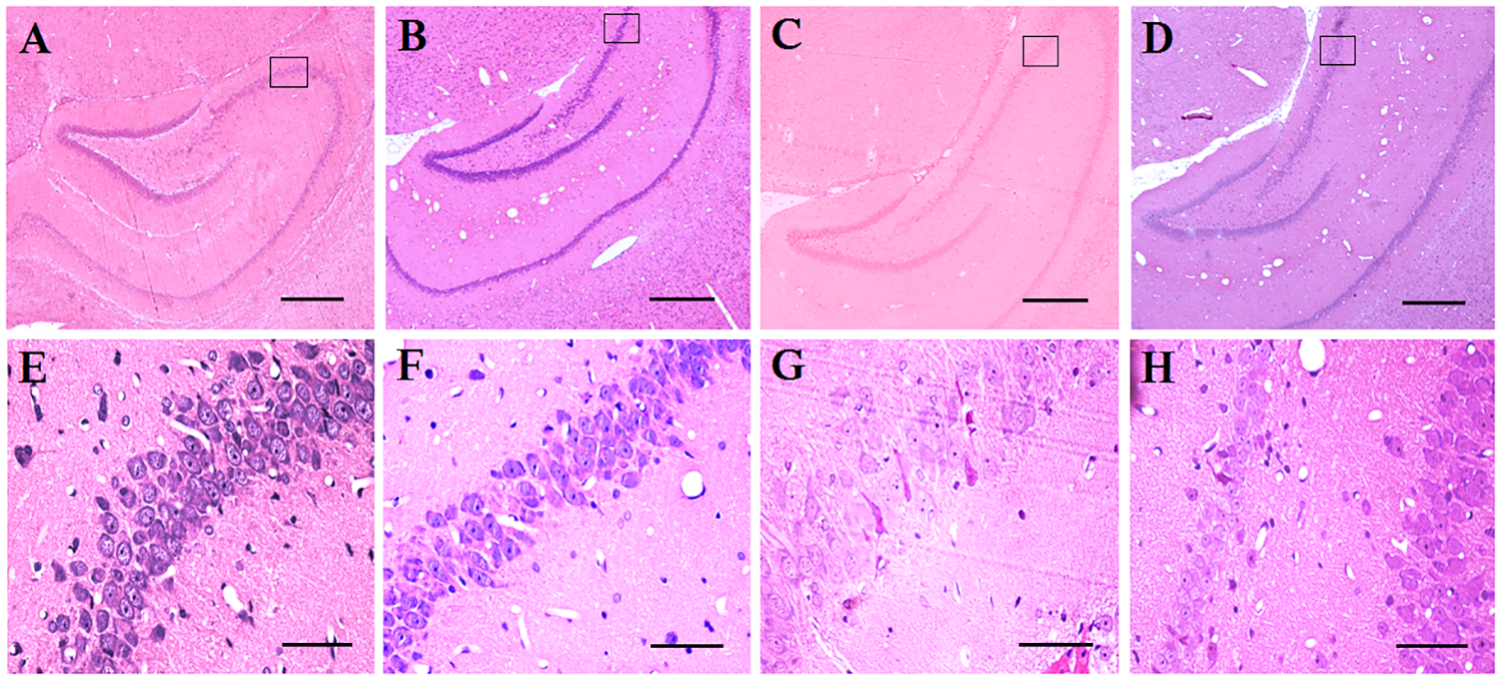
Histological examination of neuronal damage in the hippocampus of rats with treatments of control+saline, control+SGB, UCMS+saline, and UCMS+SGB (A–D, hematoxylin-eosin(HE) stain is 100 ×; E–H, HE stain is 400x). In panels A, B, C and D, boxes with letters E, F, G and H were inserted indicating the regions selected and enlarged at higher magnifications. Scale bars: A, B, C and D = 500 mm; E, F, G and H = 50 μm.

Histological analysis was used to detect neuronal damage. In this study, neuronal death and histological damage were induced by UCMS in the hippocampal CA3 region in rats. Using histological identification, in the area of hippocampal CA3, the cells showed closely arranged, prominent nuclei, with intact morphology in both sham and SGB groups, which suggests that the SGB treatment had no negative effect on neuronal structure (Fig 5A, 5B, 5E and 5F). We also found that CMS treatment resulted in more loosely arranged cells that exhibited shrinkage, partial death, and nuclear condensation, and resulted in increased neuronal loss in rats of the UCMS group (Fig 5C and 5G). However, treatment with SGB ameliorated nuclear cell shrinkage, condensation, and neuronal loss and thus provided protective effects for CMS damage (Fig 5D and 5H).

#### Effects of SGB treatment on expression of hippocampal Bcl-2 and Bax

**Fig 6:**
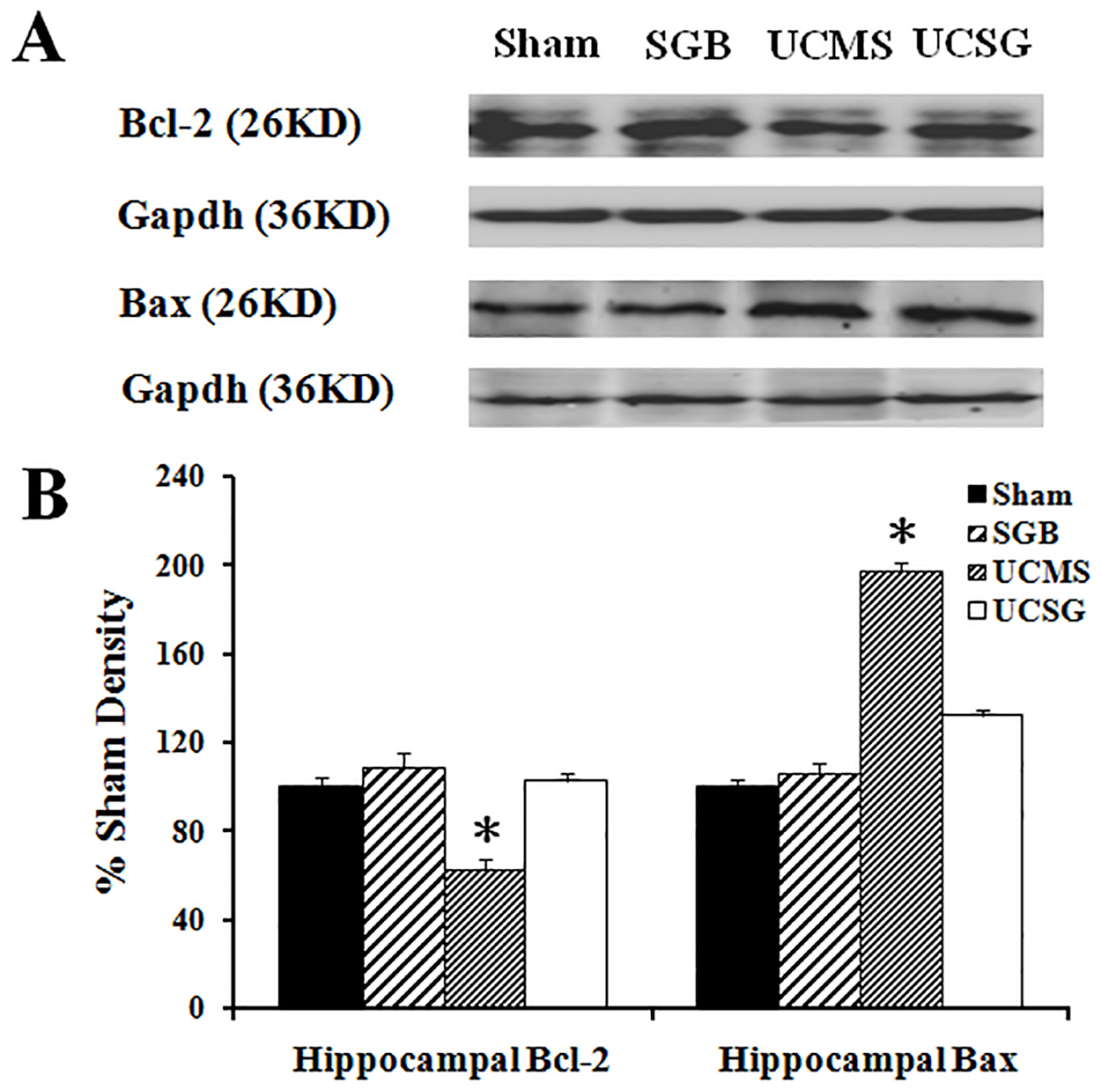
Effects of SGB on apoptotic mediators (n = 5). A, Western blot bands of hippocampal Bcl-2, Bax and GAPDH in the sham, SGB, UCMS and UCSG groups. B, Quantification of western blot bands compared with sham (100%). *, In comparison with other groups, *P* < 0.05.

In the UCMS group, there was a significant decrease in Bcl-2 and a significant increase in Bax levels in comparison to sham and SGB groups. The SGB treatment ameliorated the CMS-induced decrease in Bcl-2 and increase in Bax (*P* < 0.05; Fig 6A and 6B). These results suggest that SGB may have a neuroprotective function through upregulating Bcl-2 and downregulating Bax expression.

## Discussion

Our study has shown that SGB treatment can provide neuroprotective effects on spatial learning and memory, as well as reducing sensorimotor impairment. This finding may be explained by an anti-apoptotic mechanism, acting on two stress pathways—the HPA axis and the sympathetic-adrenomedullary system.

A good animal model is very important for investigating the pathophysiological mechanisms of depressive disorders [24]. Stress can be induced by exposure to cold, electrical shock, forced swimming, dehydration, and immobilization in animal models [25]. However, accumulating literature indicates that anhedonia induced by chronic mild stress provides a realistic animal model of depression. Such procedures are thought to adequately simulate chronic psychosocial stressors that individuals might face within society, economy, family, and human relations [19, 26]. In addition, most studies of chronic stress have focused on the effects of antidepressants and few have attempted to investigate the effects of SGB on this disorder. As mentioned previously, SGB could act to reverse the autonomic imbalance induced by increased sympathetic tone and affect the neuroendocrine system [11–13]. Moreover, bilateral SGB can lead to the increased incidence of serious complications [27]. The unilateral SGB, through systemic or local effects, can inhibit the activation of sympathetic nerve activity and regulate the HPA axis, so as to maintain or restore the stability of the autonomic nervous system [28–30]. Similarly, in clinical treatment, most of patients are treated with SGB on one side. Therefore, we used this well-established model to investigate the protective effects of right SGB on stress-induced depressive symptoms in rats.

In the present study, animals exposed to CMS for a 3-week period displayed a variety of changes in behavior, endocrine function, and neurochemistry. As previously reported, a depression-like state can be induced in the CMS model and can be measured through coat deterioration [31, 32]. Our results show that rats repeatedly exposed to mild stressors exhibited deterioration in their coat state, which was not improved despite an increase in grooming behavior. This may be due to changes in environmental conditions created by exposure to chronic stress for 21 days, such as ice-cold swimming, wet cages and cage changes, which may have contributed to the deterioration of their coat state. Sucrose consumption is considered a sensitive and specific marker of anhedonia, which is an essential component of human depression [33]. We observed that a 3-week treatment with SGB significantly improved stress-induced deterioration in coat state and restored consumption of the sucrose solution. Moreover, the reversal of these depression-like symptoms by SGB was also observed in the open-field test. The open-field test data showed that in chronically stressed rats, SGB largely restored normal movement and exploration as indicated by an increase in squares traversed and more rearing. Thus, the behavioral results from the current study indicate that the treatment of SGB had an antidepressant effect on CMS symptoms in the rat.

As an important neuroendocrine axis, the activity of the HPA axis appears to be regulated by a complex interaction of endogenous and exogenous factors [34, 35]. It plays a key role in the physiological response to stress [36]. Stress can stimulate the release of CRF from the paraventricular nucleus of the hypothalamus. The secretion of ACTH from the anterior pituitary increases in parallel with the increased release of CRF. ACTH, in turn, stimulates the release of CORT from the adrenal cortex and results in adrenal hypertrophy and hyperplasia [37]. Long-lasting HPA axis activation can disturb the balance of hormones and even cause depression [38]. The CMS exposure activates the HPA axis, and results in an increase in CORT. Long-term increases in CORT levels lead to the damage of cells in the hippocampal CA3 region, including cell shrinkage, dendrite shortening, and apoptosis. An increase in cell apoptosis in the hippocampus attenuates negative feedback inhibition of the hippocampus on the HPA axis, in turn, stimulating the release of more CORT [39]. On the other hand, a state of depression can make the HPA axis hyperactive, and alleviating clinical symptoms can have a normalizing effect on the HPA axis [40,41]. Thus, restoration of HPA axis activity may be a feasible treatment strategy for depression. In addition, chronic mild stress also activates the autonomic nervous system of the sympathetic-adrenomedullary system to increase the release of catecholamine (including norepinephrine and adrenaline). Moreover, when the sympathetic nervous system is excited, the sympathetic nerve endings can also release norepinephrine [42, 43]. In the current experiment, following a 3-week CMS treatment, the serum levels of CRF, ACTH, CORT, norepinephrine and adrenaline were elevated significantly, suggesting that CMS might lead to hyperactivity and disorder of the HPA axis and sympathetic-adrenomedullary system. However, SGB significantly reduced these stress-induced increases. After SGB treatment, the decrease in CORT in CMS-exposed rats attenuated the damage of hippocampal CA3 cells. Therefore, inhibition of the hippocampus on the HPA axis increases, and further reduces hormone levels. Such conditions are conducive to the recovery of depression-like behavior. These results indicate that SGB had an antidepressant effect by attenuating the increased levels of the hormone-regulating HPA axis and sympathetic-adrenomedullary system. These changes were consistent with the significant correlations between behavioral indexes and hormone levels.

The protective mechanisms induced by SGB were also explored by histological examinations of the hippocampus and analysis of related apoptotic proteins. In the CA3 hippocampal region, a large death of pyramidal neurons was observed following CMS. SGB treatment provided significant protective effects for this CMS damage. This finding was consistent with behavioral results from measures of spatial learning and memory and sensorimotor functioning. Apoptosis, or programmed cell death, can be regulated by caspases, tumor necrosis factor, and the Bcl-2 (B-cell CLL/lymphoma 2) family proteins. Bcl-2 and Bax are typical anti-apoptotic and pro-apoptotic members of the Bcl-2 family. Tamatani, et al. [44] indicated that the changes in Bcl-2 and Bax protein levels followed by caspase-3-like activation are a component of the cascade of nitric oxide induced neuronal apoptosis. Our results found that Bcl-2 levels were significantly decreased in the CMS-treated rats, and SGB treatment ameliorated the CMS-induced decrease. This suggests that the neuroprotective effects of SGB may have reduced neuronal apoptosis by upregulating Bcl-2. Moreover, a significant increase in Bax expression was observed in the CMS-treated rats, and SGB treatment effectively ameliorated this CMS-induced increase. Bax may therefore also play an important role in the protective effects of SGB treatment on neuronal apoptosis.

The results in the present study suggest that CMS exposure produced depression-like behavior, including anhedonia and reduced activity, in rats. Further, it raised plasma levels of CRF, ACTH, CORT, noradrenaline and adrenaline, suggesting that unpredictable chronic mild stress could enhance the activity of the HPA axis and sympathetic-adrenomedullary system, and change the expression of apoptotic proteins. SGB treatment ameliorated these depression-like changes.

## Conclusion

Our data indicate that SGB has an antidepressant effect on depression-like behaviors. The potential mechanism appears to have involved the HPA axis and sympathetic-adrenomedullary system, and had an anti-apoptotic action. The current investigation highlights a possible role for SGB as a novel supplement to traditional treatments for depression.
